# Antiretroviral regimen complexity as a predictor of adherence

**DOI:** 10.1186/1758-2652-13-S4-P121

**Published:** 2010-11-08

**Authors:** C Boyle, A Wilson, G Treacy, G Sheehan, J Lambert, C Meegan, P Mallon

**Affiliations:** 1 Mater Misericordiae University Hospital, Pharmacy Department, Dublin, Ireland; 2 Robert Gordon University, School of Computing, Aberdeen, UK; 3 Mater Misericordiae University Hospital, Department of Infectious Diseases, Dublin, Ireland; 4 University College Dublin, HIV Molecular Research Group, School of Medicine, Dublin, Ireland

## Purpose of study

Even with the introduction of once daily antiretroviral (ARV) regimens of low pill burden, long-term adherence remains a challenge, particularly in subgroups, such as patients with drug addiction. We aimed to determine levels of adherence and identify factors associated with suboptimal adherence in treated, HIV-infected patients attending a busy European inner-city HIV outpatient clinic.

## Methods

In a prospective cohort study, adherence was assessed in HIV-infected patients on antiretroviral therapy by self-report (ACTG adherence questionnaire). Relationships between suboptimal adherence (defined as <95%) and 49 covariates, including demographics, treatment factors, Centre for Epidemiological Studies Depression (CES-D) score and comorbidities were assessed using simple regression. Variables significant (P < 0.05) in univariate analyses were evaluated using multivariate logistic regression.

## Results

130 subjects (median [IQR] age 38 [11]; 27% female; 33% African origin; 27% IDU, 30% heterosexual and 20% MSM) were recruited. 83% were on once daily ARV and 16%, 34%, 48% and 2% were on regimens comprising one, two, three and four ARV medications respectively. Median CD4+ was 389 [285] cells/µL. 91% had HIV RNA < 50 copies/ml. Median adherence was 92% [range 0-100%] and 28% had suboptimal adherence. In univariate analyses, recent illicit drug use, on methadone, higher CES-D score, taking a higher number of ARV medications, greater pill burden, missed clinic appointments and lower CD4+ were associated with suboptimal adherence. In multivariate analysis, missed clinic appointments [OR 1.45; 95% CI (1.16, 1.81)] a higher CES-D score [OR 1.14; CI (1.01-1.28)] and being on a higher number of antiretroviral medications [OR 3.45; CI (1.46, 8.54)] were all independent predictors of suboptimal adherence.

## Conclusions

In a cohort where many patients are on once daily ARV, although attending clinic visits and psychological status remain important, the number of antiretroviral medications is the strongest independent predictor of adherence. Medication complexity (number of ARV) rather than the pill burden is more predictive of poor adherence in this cohort of patients from varied demographic backgrounds. Single pill, fixed dose combinations (FDC) may improve adherence and these data support further development of FDC especially for those with drug addiction and psychological issues in which current FDC medications may not be suitable.

